# Low seroprevalence of equine piroplasmosis in horses exported from the Netherlands between 2015 and 2021

**DOI:** 10.3389/fvets.2022.954046

**Published:** 2022-10-10

**Authors:** Heather Graham, Paul van Kalsbeek, Jeanet van der Goot, Miriam G. J. Koene

**Affiliations:** ^1^Department of Diagnostics and Crisis Organization, Wageningen Bioveterinary Research, Lelystad, Netherlands; ^2^Department of Bacteriology, Host Pathogen Interaction and Diagnostics, Wageningen Bioveterinary Research, Lelystad, Netherlands

**Keywords:** equine piroplasmosis, *Babesia caballi*, *Theileria equi*, seroprevalence, horses

## Abstract

Equine piroplasmosis (EP) is a tick-borne disease affecting horses, donkeys, mules and zebras, caused by the intracellular apicomplexan protozoa *Babesia caballi* and *Theileria equi*. The geographical distribution of EP is closely related to the distribution of its vector tick species belonging to the genera of *Dermacentor, Rhipicephalus* and *Hyalomma*. Since the discovery of *Dermacentor reticulatus* ticks in 2007 and the first reported autochthonous cases in the South of the Netherlands in 2012, no data on the (sero)prevalence of EP in horses in the Netherlands have been reported and it remains unclear whether *B. caballi* and *T. equi* have been able to establish themselves in the Netherlands. This study aims to give an update on the current status of EP in horses in the Netherlands using data from serological tests performed in the context of export and screening of 12,881 horses from 2015 through 2020. Horses were categorized as “Dutch,” “Foreign,” or “Unknown” based on microchip number. The overall seroprevalence of EP in Dutch horses was found to be 0.5% (95% exact CI [0.4–0.7]), compared to 1.9% (95% exact CI [1.3–2.6]) in horses in the category “Foreign” and 1.7% (95% exact CI [1.2–2.3]) in horses in the category “Unknown.” In addition, the seroprevalence per country in the category “Foreign” ranged from 0% (0.95% exact CI [0–2.8]) for Ireland to 6.0% (0.95% exact CI [3.5–9.3]) for Spain. In light of the reports on the seroprevalence during the outbreak of autochthonous EP reported in 2012 and on seroprevalences of EP in other countries in Northwestern Europe, the seroprevalence of EP in horses exported from the Netherlands is very low. However, the higher seroprevalence of EP in horses from abroad warrants the need for the monitoring of EP, as tick vectors are present in the Netherlands and the import of horses from endemic areas increases the chances of EP becoming more prevalent in the Netherlands.

## Introduction

Equine piroplasmosis (EP), also known as equine babesiosis or biliary fever, is a tick-borne disease affecting horses, donkeys, mules and zebras, caused by the intracellular apicomplexan protozoa *Babesia caballi* and *Theileria equi* (formerly *Babesia equi*), either individually or in the form of a co-infection (1, 2). EP is transmitted by tick vectors or iatrogenically (for instance, through blood transfusion or contaminated needles), while transplacental transmission has also been described (1, 3). Tick species belonging to the genera of *Dermacentor, Rhipicephalus* and *Hyalomma* are considered to be competent vectors, but the process of identifying other competent tick vectors is still ongoing (1, 4). For *B. caballi*, infected equids as well as tick vectors act as a reservoir, while only chronically infected equids are considered as the reservoir for *T. equi* (2).

Clinical symptoms of EP include signs of hemolytic anemia, such as icterus and hemoglobinuria, fever and signs of systemic illness (1, 2). Apparently healthy mares can transmit *T. equi* to the unborn foal, leading to abortion or neonatal piroplasmosis (1, 2). The infection manifests in several forms: peracute, acute, subacute or chronic (2, 4). In the case of *T. equi*, the persistent infection is life-long, while for *B. caballi*, evidence exists that some infected horses are able to clear the parasite (2). In areas where EP is considered to be endemic, clinical manifestation during outbreaks is rare and the majority of infected horses are believed to become inapparent carriers (2). However, depending on factors like immunity, the mortality of horses in endemic areas can be as high as 5–10% (2, 5). Peracute or acute symptoms of EP usually derive from the introduction of naive horses in endemic areas, especially when ticks are abundant (2). In non-endemic areas, EP can result in the death of up to 50% of the infected horses, while most horses that survive infection, become asymptomatic carriers, similar to situations where EP is endemic (2, 5). Asymptomatic carriers serve as a source of infection for tick vectors, but are also at risk of relapse, for instance, after demanding exercise (5, 6). Apart from the impact on animal health and welfare, EP leads to substantial economic losses in the equine sector (5). Treatment of EP is difficult and often without success. Imidocarb dipropionate has proven to be effective in reducing clinical symptoms in several studies, but complete clearance of the parasite, especially *T. equi* remains a challenge (6–8). To date, there is no vaccine commercially available. Strategies aiming at prevention of infection include tick control measures, such as the application of tick-specific repellents and trade restrictions, mainly in the form of required health certificates stating the animals are free of EP.

The geographical distribution of EP is closely related to the distribution of its vector tick species belonging to the genera of *Dermacentor, Rhipicephalus* and *Hyalomma* which are endemic in tropical and subtropical regions, hence the majority of cases of EP are reported in Africa, Asia, Central and South America and southern parts of Europa and the USA (1, 2, 4). When tick vectors are present in a particular country or region considered to be free of EP, the introduction of an infected horse can result in the epizootic spread of EP. This is why the global movement of horses for trade and equine events poses a risk for non-endemic nations. Historically, the Netherlands were considered to be free of EP and its tick vectors were reported only sporadically or not at all. However, over the last few years, *Dermacentor* ticks have been found more frequently in regions with a more moderate climate (9, 10). In 2007, the presence of a population of *Dermacentor reticulatus* ticks in the Netherlands was described for the first time (11). *Dermacentor reticulatus* had previously only been detected on imported animals (11). A few years later, DNA of *B. caballi* and *T. equi* was isolated for the first time from ticks collected from horses in the Netherlands between 2008 and 2009 (12). Following the discovery of both of these agents responsible for EP, a horse with a subclinical *B. caballi* infection was presented to a veterinary clinic in the Netherlands in 2009 (13). The horse had never been abroad and this case led to a seroprevalence survey in an area in the South West of the Netherlands, where the *B. caballi* infected horse was kept (13). Twelve (4%) of the 300 randomly selected horses in the area were seropositive for EP, while five (1.6%) tested positive for *T. equi* DNA by performing polymerase chain reaction combined with reverse line blotting (PCR-RLB) (13). Of these positive horses, four (1.3%) had never left the country, providing evidence for autochthonous EP infections in the Netherlands. During this survey, the researchers were contacted about two indigenous horses with clinical signs of EP outside of the sampling area, which were later diagnosed with an acute *T. equi* infection (13). These horses with *B. caballi* and *T. equi* infections were the first reported autochthonous cases of EP in the Netherlands.

Since then, no data on the (sero)prevalence of EP in horses in the Netherlands has been reported. It remains unclear whether *B. caballi* and *T. equi* have been able to establish themselves in the Netherlands since the first reports of autochthonous cases of EP in 2012 and how the current seroprevalence of EP in apparently healthy horses compares to the 4% reported during the outbreak in 2009 and 2010 (13). EP is not notifiable in the Netherlands and there is no surveillance program in place. This study aims to give an update on the current status of EP in horses in the Netherlands using data from serological tests performed at the national reference laboratory (NRL) in the context of export and screening between 2015 and 2021. In addition, a distinction was made between Dutch horses and horses from abroad based on microchip number to be able to compare the seroprevalence of EP in the indigenous equine population to the seroprevalence of EP in imported horses.

## Materials and methods

### Origin of samples

Being the Dutch NRL for export diagnostics, Wageningen Bioveterinary Research (WBVR) receives sera from equine export and transport companies, either directly or through a veterinary practice. In most cases, importing countries will request one or more specific tests to clear an animal for import. As a result, some horses are tested by indirect fluorescence antibody test (IFAT) only, others by competitive enzym-linked immunosorbent assay (cELISA) and IFAT, some by cELISA, IFAT and complement fixation test (CFT), and so on. In addition, sera are sent to the NRL by equine breeding companies to maintain a certain health status. Finally, serum samples are submitted for screening purposes, for example, prior to participation in equestrian competitions. Test results are entered in a digital database and as such, were available for analysis. It should be noted that the majority (>90%) of the samples included in this study, were tested for the purpose of export of the animal. This means that the majority of the horses included in this study, resided in the Netherlands for at least the length of the period of quarantine required for export and that they did not show any clinical symptoms when the samples were collected.

### Serological tests

All serological tests were performed at the NRL and in parallel for each sample. The IFAT was performed using an in-house protocol in accordance with the OIE manual and slides provided by the Faculty of Veterinary Medicine in Utrecht, The Netherlands from 2015 up to and including the first half of 2018 (14). From the second half of 2018 up to 2020, the IFAT was performed using an in-house protocol in accordance with the OIE manual and commercial slides (MegaFLUO^®^ BABESIA caballi and MegaFLUO^®^ THEILERIA equi, MEGACOR, Diagnostik GmbH, Hörbranz, Austria) (14). Samples with a titer of 80 or higher were considered positive. The exact sensitivity and specificity of the IFAT are unknown, but the IFAT is considered as a highly specific confirmatory test (15). For this study, the specificity of the IFAT was assumed to be 100%.

The CFT was performed using an in-house protocol in accordance with the OIE manual. Samples with a titer of 5 or higher were considered positive. The exact sensitivity and specificity of the CFT performed in-house are unknown, but the CFT in general is considered to be less sensitive than the cELISA (15). For this study, the specificity of the CFT was assumed to be 100%.

Commercial cELISA kits for *B. caballi* (*Babesia caballi* Antibody Test Kit, cELISA, VMRD, USA) and *T. equi* (*Theileria equi* Antibody Test Kit, cELISA, VMRD, USA) were used to test the submitted samples by cELISA maintaining the cut-off value of ≥40% as described by the kit manuals. The cELISA is considered to be a highly sensitive as well as a highly specific test according to the OIE manual: the specificity appears to be 99.5% for *B. caballi* and 99.2% for *T. equi* (14). However, for this study, the specificity of both cELISAs was assumed to be 100%.

### Sample selection and analysis of results

For this study, data was checked and filtered based on a number of conditions. Test results deriving from samples that had been tested by IFAT, cELISA and/or CFT for both *B. caballi* and *T. equi* between 2015 and 2020 were included. Occasionally, multiple samples from one individual horse are submitted within a few weeks or months or over the years. As this study focuses on individual horses rather than samples, the decision was made to only include the (chronologically) first test result of every horse, that had been entered in the database between 2015 and 2020. Consequently, a positive result following a negative result was not taken into account (and vice versa). Furthermore, a horse was considered to be seropositive if one or more serological tests yielded a positive result, disregarding the negative results of other tests when performed. Only results from horses and no other animal species were taken into account. As this study aims to represent the (healthy) equine population in the Netherlands, the reason for submission had to be either export or screening in order to exclude any clinical cases. Finally, horses were divided into three categories based on the microchip numbers as stated on the submission forms: “Dutch” (valid microchip numbers starting with 528), “Foreign” (valid microchip numbers starting with anything other than 528 and up to and excluding 900) and “Unknown” (invalid microchip numbers, names, no information, microchip numbers starting from 900). Microchip numbers starting from 900 do not refer to a specific country, but are property of the microchip manufacturer.

## Results

### Seroprevalence of EP per category

A total of 52,219 serological test results were entered in the database between 2015 and 2020. Filtering as described above, resulted in a total of 12,881 unique animals tested for antibodies against both *B. caballi* and *T. equi*, of which 9,148 (71%) animals belonged to the category “Dutch,” 1,611 (13%) to the category “Foreign” and 2,122 (16%) to the category “Unknown”.

Concerning horses in the category “Dutch”, 22 out of 9,148 horses (0.2%, 95% exact CI [0.2–0.4]) tested positive according to at least one serological test for *B. caballi*, compared to 27 out of 9,148 (0.3%, 95% exact CI [0.2–0.4]) for *T. equi* (Table 1). Four horses tested positive for both *B. caballi* as well as *T. equi* antibodies resulting in an overall seroprevalence of EP of 0.5% (95% exact CI [0.4–0.7]) (Table 1).

**Table 1:** Total numbers of seropositive horses for *B. caballi, T. equi* and combined (“EP seropositive”) compared to the total of horses tested between 2015 through to 2020 per category based on microchip numbers (“Dutch,” “Foreign” and “Unknown”).

**Category**	**Dutch**		**Foreign**		**Unknown**	
Number of *B. caballi* seropositive horses/total number of tested horses	22/9148	0.2% [0.2–0.4]	9/1611	0.6% [0.3–1.1]	8/2122	0.4% [0.2–0.7]
Number of *T. equi* seropositive horses/total number of tested horses	27/9148	0.3% [0.2–0.4]	25/1611	1.6% [1.0–2.3]	30/2122	1.4% [1.0–2.0]
Total number of EP seropositive horses/ total number of tested horses	45/9148	0.5% [0.4–0.7]	30/1611	1.9% [1.3–2.6]	36/2122	1.7% [1.2–2.3]

Seroprevalences are also given as percentages including the 95% exact confidence interval in square brackets.

In the category “Foreign,” nine out of 1,611 horses (0.6%, 95% exact CI [0.3–1.1]) tested positive according to at least one serological test for *B. caballi*, compared to 25 out of 1,611 (1.6%, 95% exact CI [1.0–2.3]) for *T. equi* (Table 1). Four horses tested positive for both *B. caballi* antibodies as well as *T. equi* antibodies resulting in an overall seroprevalence of EP of 1.9% (95% exact CI [1.3–2.6]) (Table 1).

**Figure 1:**
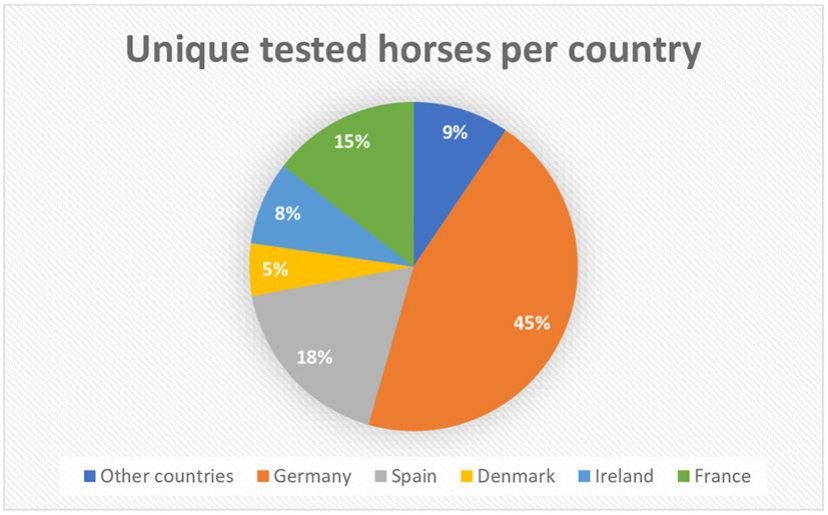
Distribution of the origin of horses in the category “Foreign” (*n* = 1,611), based on microchip numbers and country codes. 91% (*n* = 1,466) of the horses originate from Germany, Spain, France, Ireland, and Denmark, constituting the top five, while 9% (*n* = 145) of the horses in the category “Foreign” originate from other countries.

Based on the microchip numbers of the horses stated on the submission forms, the origin of the horses in the category “Foreign” could be determined. Of the 1,611 horses in this category, 45% of the horses were microchipped with a German microchip number, 18% with a Spanish microchip number, 15% with a French microchip number, 8% with an Irish microchip number and 5% with a Danish microchip number (Figure 1). The percentages for the other countries (13 countries in total) were below 5% and were grouped together under “Other countries,” making up 9% of the horses in the category “Foreign” (Figure 1). The seroprevalences of EP in the countries making up 91% of the submissions are shown in Table 2. The highest seroprevalence (6.0%, 95% exact CI [3.5–9.3]) was found in horses originating from Spain, followed by France (1.7%, 95% exact CI [0.5–4.3]) and Germany (0.6%, 95% exact CI [0.2–1.4]). The dataset did not include any seropositive animals with a Danish or an Irish microchip number. The seoprevalence of EP for the grouped other countries was (3.3%, 95% exact CI [1.1–7.5]).

**Table 2:** Total numbers of seropositive horses for *B. caballi, T. equi* and combined (“EP seropositive”) compared to the total of horses tested between 2015 through to 2020 per category based on microchip numbers and country codes.

**Country of origin**	**Germany**		**Spain**		**France**	
Number of *B. caballi* seropositive horses/total number of tested horses	1/724	0.1% [0.003–0.8]	5/285	1.8% [0.6–4.0%]	2/234	0.9% [0.1–1.1]
Number of *T. equi* seropositive horses/total number of tested horses	3/724	0.4% [0.09–1.0]	14/285	4.9% [2.7–8.1]	3/234	1.3% [0.3–3.7]
Total number of EP seropositive horses/ total number of tested horses	4/724	0.6% [0.2–1.4]	17/285	6.0% [3.5–9.3]	4/234	1.7% [0.5–4.3]
**Country of origin**	**Ireland**		**Denmark**		**Other countries**	
Number of *B. caballi* seropositive horses/total number of tested horses	0/132	0% [0–2.8]	0/83	0% [0–4.3]	1/153	0.7% [0.02–3.6]
Number of *T. equi* seropositive horses/total number of tested horses	0/132	0% [0–2.8]	0/83	0% [0–4.3]	4/153	2.6% [0.7–6.6]
Total number of EP seropositive horses/ total number of tested horses	0/132	0% [0–2.8]	0/83	0% [0–4.3]	5/153	3.3% [1.1–7.5]

Seroprevalences are also given as percentages including the 95% exact confidence interval in square brackets.

As for the horses in the category “Unknown,” eight out of 2,122 horses (0.4%, 95% exact CI [0.2–0.7]) tested positive according to at least one serological test for *B. caballi*, compared to 30 out of 2,122 horses (1.4%, 95% exact CI [1.0–2.0]) for *T. equi* (Table 1). Two horses tested positive for both B. *caballi* antibodies as well as *T. equi* antibodies resulting in an overall seroprevalence of EP of 1.7% (95% exact CI [1.2–2.3]) (Table 1).

## Discussion

The aim of this study was to provide an update of the current status of EP in the Netherlands based on the data of routine diagnostics for export, since no data on the (sero)prevalence of EP has been published since the first infections with EP in autochthonous horses in 2009 (13). The overall seroprevalence of EP in healthy Dutch horses was calculated to be 0.5% (95% exact CI [0.4–0.7]) based on the data of routine diagnostics for export between 2015 and 2020, compared to the seroprevalence of 4% found during the survey in a local area where autochthonous cases of EP were reported in 2012 (13). Despite the differences between these studies, namely 300 horses in the vicinity of autochthonous cases of EP, having access to pasture and/or being used for outdoor recreation, and sampled in the summer months of 2010 vs. 9148 healthy (indigenous) horses sampled between 2015 and 2021 in the context of export and screening, the seroprevalence of 0.5% in Dutch horses as found by this study a decade later, does not indicate that EP is a disease of significance in the Netherlands. It may be argued that the true seroprevalence of EP in the Netherlands possibly exceeds this percentage, as the data used for this study mainly derives from horses intended for export. This presumably involves above-average care and indoor-housing, thus reducing the probability of contracting EP. However, EP is also rarely diagnosed in the field and usually involves imported horses from endemic areas (C. van Maanen, personal communication, January 10^th^ 2022). This supports the low seroprevalence as found by this study and there is no indication that the true seroprevalence will be much higher. However, studies including horses not intended for export should be carried out to establish how the prevalence found in this study relates to the true seroprevalence.

During the survey following the first autochthonous case in 2009, two ponies from outside the sampling area were diagnosed with acute EP and were considered to be indigenous as well (13). This implies that competent tick vectors for the transmission of EP were present in at least part of the Netherlands. This was confirmed by a study that reported the detection of DNA of both *B. caballi* and *T. equi* from *Ixodes ricinus* ticks collected from Dutch horses in 2008–2009 (12). It should be noted that *I. ricinus* is not considered as a typical vector for EP, but its capability of transmitting either *B. caballi* or *T. equi* could have implications for countries where this tick species is abundant (16). Since the outbreak of EP as described by Butler et al. (13), three surveys have been published that have investigated the presence of EP tick vectors and the presence of *B. caballi* or *T. equi* in those tick vectors in the Netherlands. Jongejan et al. (17) analyzed 855 *D. reticulatus* ticks from various sites in the Netherlands (*n* = 566) and Belgium (*n* = 289) by PCR/RLB, of which one tick from the Netherlands and one tick from Belgium tested positive for *B. caballi* (17). No ticks tested positive for *T. equi* (17). In another study in 2019, none of 860 *D. reticulatus* ticks collected in the Netherlands were found to be positive for either *B. caballi* or *T. equi* by means of a high-throughput real-time PCR based array (18). In another more recent study in 2021, 17 *Hyalomma* ticks, of which 15 discovered on horses in the Netherlands were analyzed for the presence of pathogens, such as *B. caballi* and *T. equi* (19). All *Hyalomma* ticks, which were most likely introductions, tested negative for the presence of EP parasites by PCR, and an additional cross-sectional study in 202 horses in 2019 revealed that *Hyalomma* ticks could not be detected on these horses during the observatory period (19). The very low prevalence of *B. caballi* and *T. equi* in the Dutch tick population including introductions seems to support the low seroprevalence found in Dutch horses intended for export.

The overall seroprevalence of EP in horses from abroad (category “Foreign”) based on the data from 2015 through 2020 was calculated to be 1.9% (95% exact CI [1.3–2.6]), which is higher than the seroprevalence of EP in Dutch horses (0.5% (95% exact CI [0.4–0.7])). The highest seroprevalences of 6.0% (95% exact CI [3.5–9.3]) and 1.7% (95% exact CI [0.5–4.3]) were found in horses originating from, respectively, Spain and France, which is to be expected, as EP is considered to be endemic in Spain and (some parts of) France (4, 20). Being a neighboring country, it is not surprising that the seroprevalence of horses originating from Germany (0.6% (95% exact CI [0.2–1.4]) is more in line with the seroprevalence of Dutch horses (0.5% (95% exact CI [0.4–0.7]).

As the category “Unknown” may include both autochthonous as well as non-autochthonous horses, the percentages are difficult to interpret. However, given the probable mix in origin of the animals in this category, it seems logical that the overall seroprevalence of 1.7% (95% exact CI [1.2–2.3]) lies somewhere in between the seroprevalences of EP in the categories “Foreign” and “Dutch.”

Studies similar to this have been conducted in other countries in Northwestern Europe. A recent study from the United Kingdom (UK) revealed seroprevalences of 4.4% for *B. caballi*, 5.9% for *T. equi* and an overall seroprevalence of EP of 8.0% (21). These seroprevalences are considerably higher than our observations, while both studies make use of samples submitted for routine diagnostics in the context of export, and are more in the line of the seroprevalence reported in Switzerland (7.3%) (22). A similar study with samples submitted for screening in Ireland showed an overall seroprevalence of 3.5% (1.5% for *B. caballi* and 2.5% for *T. equi*) which is more comparable to the seroprevalences found in our study compared to the seroprevalence of 8.0% in the UK (23). Due to legislation concerning data protection, no detailed information was available on the origin of the tested horses (23). However, the seroprevalence is still at least twice as high as the seroprevalences in both Dutch horses and horses originating from abroad in our study. This also holds true for the seroprevalence of 6.1% for *T. equi* reported by researchers in Germany, where 314 indigenous horses were tested for EP (24). In contrast, the researchers also report a seroprevalence of 0.3% for *B. caballi*, which is more in line with our findings, including both the seroprevalence in Dutch horses of 0.2% (95% exact CI [0.2–0.4]) as well as the seroprevalence found in German horses of 0.1% (95% exact CI [0.003–0.8]) (24).

The difference between the results of our study and the studies conducted in the UK and Ireland can at least partially be explained by our decision to analyze at the level of individual horses rather than individual samples. While analyzing the data, it became clear to us that multiple submissions within weeks or months after the first positive test result were not uncommon. This can be explained by the nature of the samples, which were mainly submitted to meet the health requirements of importing countries. The antibody titer of a seropositive animal will likely decline over time and there is a probability that the seropositive animal will test negative at some point, which means that the animal can be exported. Some horses in our dataset were tested positive up to three times on multiple occasions following an initial positive test result. The great majority of tested horses is assumed to be exported when tested negative and there is no reason for a new sample to be submitted. Indeed, the pattern of multiple submissions was observed to a far lesser extent for horses that were tested negative the first time as opposed to horses that were tested positive. To avoid the potential bias and overestimation of the seroprevalence caused by the consecutively submitted, seropositive samples, only the (chronologically) first test result of a horse that had been entered in the database was taken into account in our study.

Another factor that might explain the differences in seroprevalences to some extent might be the application of different serological tests between the studies. As described earlier, the sensitivity differs between the serological tests. This is demonstrated by the study in Germany, where the only positive *B. caballi* cELISA result could not be confirmed by IFAT and only 10 out of 16 positive *T. equi* IFAT results could be confirmed by cELISA (24). The difference in sensitivity of the applied tests is one of the reasons that for this study a horse was considered to be seropositive if the result of at least one of the serological tests was defined as positive, disregarding the negative results of other tests for that submission when performed. In addition, the specificity of all tests was assumed to be 100%.

Another factor that may have led to a potential underestimation of the seroprevalence of EP in this study is the pre-screening of horses. For official reports (recognized by the Dutch authorities), export companies or horse owners are required to submit their samples to the NRL in the Netherlands. However, they can have their horses tested by another laboratory when an official report is not necessary, allowing them to get insight in the serological status of the animals and exclude seropositive horses from export. It is unknown at which scale this pre-screening takes place and how much this bias will have affected the results.

The very low seroprevalence of EP in Dutch horses in this study is in accordance with the very low prevalence of *B. caballi* and *T. equi* in the Dutch tick population: it seems that little over a decade after the first reported outbreak of EP infections in autochthonous horses, the seroprevalence of EP in the Netherlands is still very low. The latter is especially true for supposedly indigenous horses and compared to other countries in Northwestern Europe. Whether the very low seroprevalence in horses tested for the purpose of export is representative for the seroprevalence in the field needs to be confirmed. It is left to speculation to which extent regional differences in tick distribution, climate or other factors account for the differences between seroprevalence studies conducted in several countries in Northwestern Europe. Nevertheless, suitable tick vectors for EP are present in the Netherlands and based on the higher seroprevalence of horses imported from endemic areas, as shown by the results of this study, it is recommended to continue monitoring the status of EP in both tick vectors and horses in the Netherlands.

## Data availability statement

The raw data supporting the conclusions of this article will be made available by the authors, without undue reservation.

## Ethics statement

Ethical review and approval was not required for the animal study because the study used data on previously tested animals of which samples were submitted in the context of export and screening.

## Author contributions

HG initiated and designed the study and wrote the first draft of the manuscript. PK performed the analysis of the data. JG supervised the design of the study, data analysis, and statistical analysis of the data. HG, PK, JG, and MK were actively involved in the interpretation of the data. JG and MK reviewed the manuscript. All authors read and approved the final version of the manuscript.

## Funding

This study was primarily financed by the Ministry of Agriculture, Nature and Food Quality through an ongoing project on equine diseases within the framework of statutory tasks, Grant Number WOT-01-002-005.03.

## Conflict of interest

The authors declare that the research was conducted in the absence of any commercial or financial relationships that could be construed as a potential conflict of interest.

## Publisher's note

All claims expressed in this article are solely those of the authors and do not necessarily represent those of their affiliated organizations, or those of the publisher, the editors and the reviewers. Any product that may be evaluated in this article, or claim that may be made by its manufacturer, is not guaranteed or endorsed by the publisher.
